# Health survey on anxiety, depression, and stress in Afghanistan: A large-scale cross-sectional study amid ongoing challenges

**DOI:** 10.1007/s44192-024-00090-5

**Published:** 2024-09-20

**Authors:** Ahmad Neyazi, Abdul Qadim Mohammadi, Nosaibah Razaqi, Bilal Ahmad Rahimi, Sifatullah Sifat, Najeebullah Rahimy, Zarghoon Tareen, Qasim Mehmood, Prakasini Satapathy, Mark D. Griffiths

**Affiliations:** 1 Afghanistan Center for Epidemiological Studies, Herat, 3001 Afghanistan; 2 Department of Mental Health, Herat Regional Hospital, Herat, Afghanistan; 3 Department of Pediatrics, Kandahar University, Kandahar, Afghanistan; 4 Dr Shams-ul-Haq Kakar Comprehensive Health Center, Kandahar, Afghanistan; 5 Department of Histopathology, Kandahar University, Kandahar, Afghanistan; 6 King Edward Medical University, Lahore, Pakistan; 7 Center for Global Health Research, Saveetha Medical College and Hospital, Saveetha Institute of Medical and Technical Sciences, Saveetha University, Chennai, India; 8 Department of Psychology, Nottingham Trent University, Nottingham, UK; 9 Faculty of Medicine, Ghalib University, Herat, Afghanistan

**Keywords:** Depression, Anxiety, Stress, Afghanistan, Taliban

## Abstract

**Background:**

One of the most significant events in recent Afghan history is the rise of the Taliban and the war that followed, which had profound impacts on the lives of Afghans. The present study examined the mental health of Afghans living under the Taliban government.

**Methods:**

Between June 5, 2023 and February 12, 2024, a cross-sectional study was conducted among the Afghan population in three key regions of Afghanistan. Data were collected using a pre-tested structured questionnaire. The 21-item Depression, Anxiety and Stress scale (DASS-21) was utilized to assess depression, anxiety and stress of the Afghan population. Ethical permission for this study was granted by the Afghanistan Center for Epidemiological Studies (ACES). Logistic regression models were employed to explore the relationship between socio-demographic characteristics and depression, anxiety, and stress among 2,698 participants.

**Results:**

The prevalence of depression was 72.05%, anxiety was 71.94%, and stress was 66.49%. Multiple regression analysis indicated that gender (being female), economic status (being poor), residency (living in rural areas), education level (being illiterate), being a cigarette smoker, and having experienced a bad event during the past month were significantly associated with depression, anxiety and stress.

**Conclusion:**

The findings of the present study show very high levels of anxiety, depression, and stress, most likely reflecting the profound impact of recent political, social, and economic changes. Notably, a significant majority of participants, particularly females and individuals above 35 years of age, reported severe to extremely severe mental health symptoms. The mental health crisis in Afghanistan is a complex and urgent issue that requires a comprehensive and compassionate response.

## Introduction

Afghanistan, located in South Asia, has been a region of significant political and geopolitical importance for decades. The country has experienced a tumultuous history, marked by conflicts, foreign interventions, and political changes [1]. One of the most significant events in recent Afghan history is the rise of the Taliban and the war that followed, which had profound impacts on the lives of Afghans [1, 2].

The Taliban is an extremist Islamist group that emerged in the early 1990s. They gained control of Afghanistan in 1996, imposing a strict interpretation of Islamic law. Following the 9/11 attacks in the United States in 2001, the U.S. and its allies initiated Operation Enduring Freedom to oust the Taliban regime and dismantle Al-Qaeda [3, 4]. The war evolved into a protracted conflict, characterized by insurgency, counterinsurgency efforts, and complex alliances with Afghan factions [5].

After the fall of the Taliban in 2001, efforts were made to rebuild Afghanistan, establish democratic institutions, and promote human rights and gender equality. Despite these efforts, corruption, political instability, and a resurgent Taliban posed ongoing challenges to the nation's development. However, in August 2021, the Taliban regained control of the country as United States and NATO (North Atlantic Treaty Organization) forces withdrew, leading to concerns about the protection of human rights and the Afghan people's future [3, 5].

The Afghan conflict has had far-reaching global implications, influencing regional stability, refugee crises, and counterterrorism efforts [6]. The situation in Afghanistan is highly fluid, and the well-being of its citizens remain a global concern. The complex history of Afghanistan, the rise of the Taliban, and the enduring war have left a deep impact on the lives of Afghans, shaping the country's destiny and its place in the world [6, 7].

The mental health of the Afghan people has long been a matter of concern due to decades of conflict and instability in the region. The return of the Taliban to power in Afghanistan in recent years has raised new and urgent questions about the well-being of its citizens [8]. Afghanistan has been marred by war and violence for generations. The resurgence of the Taliban has the potential to trigger or exacerbate trauma among individuals who have experienced violence, displacement, and loss [8, 9]. Post-traumatic stress disorder (PTSD) is a significant concern, given the cumulative effects of prolonged conflict [10].

The uncertainty associated with Taliban rule, especially concerning women's rights, freedom of expression, and personal freedoms, appears to have generated widespread anxiety and fear among Afghan citizens [10, 11]. Living under strict rules and facing harsh consequences for non-compliance can take a significant toll on individuals’ mental well-being. The loss of livelihoods, the breakdown of essential services, and the general sense of hopelessness can lead to high rates of depression among the Afghan population [10, 12]. Furthermore, the lack of economic prospects and the absence of a stable environment can contribute to a sense of despair [12].

Mental health issues are often stigmatized in Afghan society, making it challenging for individuals to seek help or confide in others [13, 14]. The fear of being ostracized or labeled as weak can lead to social isolation for those grappling with mental health challenges [15, 16]. Also, the country is facing significant hurdles in providing mental health services to the Afghan people. Conflict and instability have strained the healthcare system, making it difficult for individuals to access the support they need. The current situation has likely exacerbated this issue, leaving many without proper care [17, 18]. Furthermore, the condition of Afghan women under Taliban rule cannot be underestimated. Gender-based violence, forced marriages, and the fear of losing access to education and employment can lead to severe anxiety, depression, and trauma among women [9, 19].

With approximately 90% of the Afghan population experiencing the deleterious effects of poverty [20], the plight of women and girls has become even more pronounced [21]. Under the governance of the Taliban, women encounter constraints in various domains, including education, employment, mobility, political involvement, healthcare access, and public visibility [21]. These circumstances have the potential to exacerbate pre-existing mental health disorders among the populace. Additionally, the advent of COVID-19, coupled with other contributing factors (e.g., poverty), is likely to have adversely affected mental well-being [22]. The closure of numerous schools and daycare facilities has compelled women to shoulder additional responsibilities, including the care of children and/or elderly family members, often concurrently managing remote work obligations [21]. This heightened workload has the potential to engender feelings of fatigue, frustration, and burnout, collectively posing a considerable threat to mental health [23].

Since the Taliban regained control of Afghanistan, the health standards among the Afghan population have deteriorated significantly. The impact of the Taliban's governance, along with the influence of extremist groups such as ISIS (Islamic State of Iraq and Syria), has contributed to a notable decline in mental health. The high prevalence of anxiety, depression, and stress among these groups, often referred to as 'ISIS syndrome,' suggests that the psychological state of these extremists could be a factor in the worsening mental health crisis in Afghanistan [24, 25].

Despite the aforementioned research, there has been no prior research examining the levels of depression, anxiety, and stress experienced by Afghans at a national level during the Taliban's governance. Therefore, the present study examined these mental health aspects collectively among Afghans. Additionally, it explored the underlying factors associated with depression, anxiety, and stress. Lastly, the study examined how socio-demographic traits of Afghans under Taliban rule relate to these three mental health indicators. As the study was exploratory, there were no specific hypotheses.

## Methods

### Participants, study design, and procedure

**Fig. 1:**
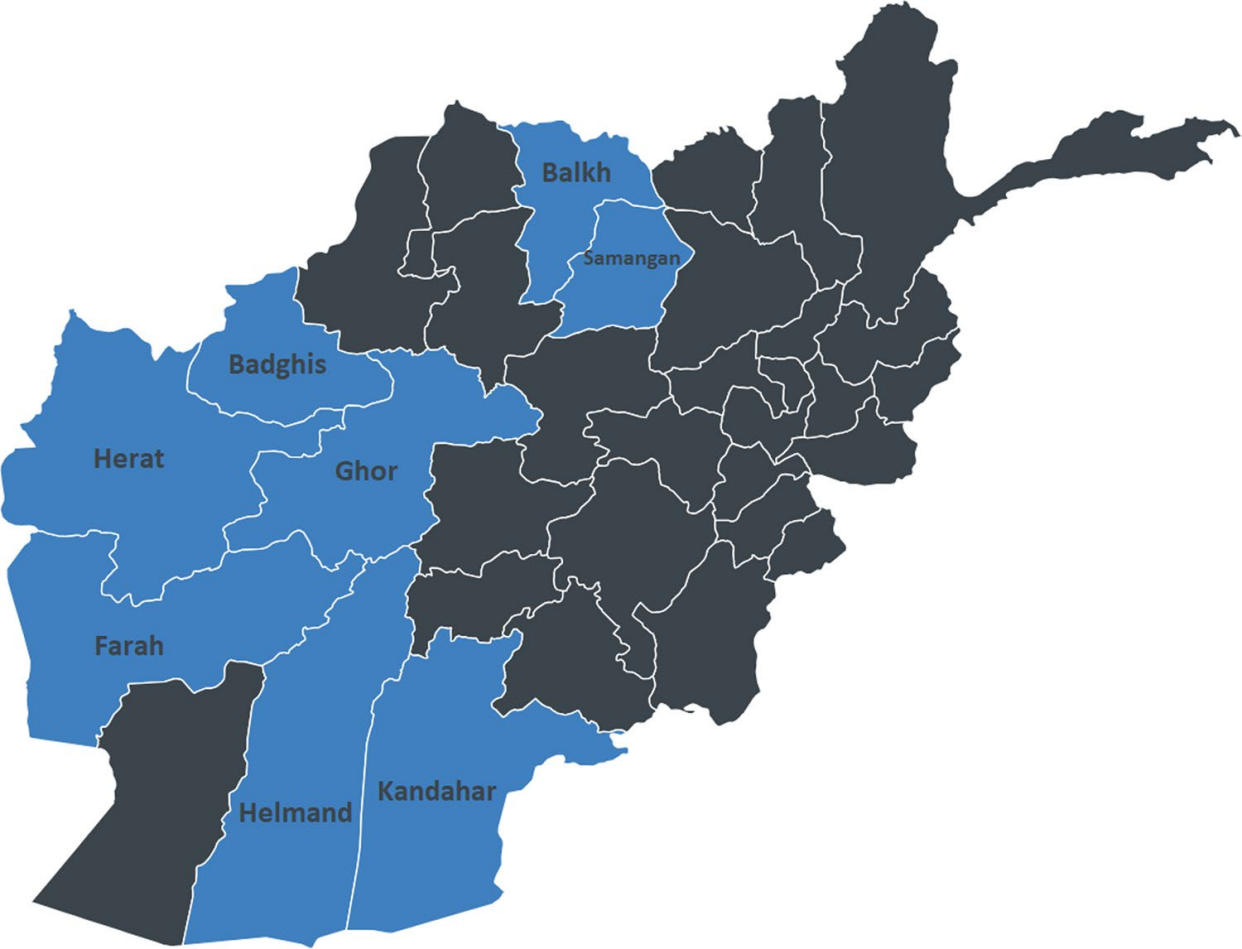
Provinces included in the present study. (The blue color denotes the provinces included in the study, while the black color indicates the provinces where data collection was not carried out)

Between June 5, 2023 and February 12, 2024, a cross-sectional study was conducted. The study comprised 2,698 participants (1234 men and 1464 women), ranging in age from 15 to 100 years (mean age = 30.96 years; SD ± 13.70). These participants were recruited from various regions in Afghanistan, namely the southern region (Kandahar, Helmand), western region (Herat, Badghis, Farah, Ghor), and northern region (Mazar-e-Sharif, Samangan) (Fig. 1). Data were collected through face-to-face interviews conducted by 15 trained data collectors. Participants were recruited using cluster convenience sampling. A total of 3080 individuals residing in the aforementioned provinces were directly invited to participate in the study by approaching them outdoors in streets where they lived and/or worked. Among these, 2698 individuals volunteered to be interviewed (response rate = 87.6%).

To be eligible for the study, individuals had to meet specific criteria. More specifically, they had to be: (i) residents of Afghanistan, (ii) aged at least 15 years, and (iii) able to understand either Dari or Pashto languages, and (iv) able to provide either written or verbal informed consent (those aged 15–18 years were also required to have additional consent from their parents). The target sample size was determined using the formula N = Zα2P(1 − P)/d2, with α = 0.05 and Zα = 1.96, and a margin of error (d) of 5% which means the minimum sample size for each cluster (province) was 385. The proportion of women experiencing depression and anxiety was estimated at 80% based on existing Afghan literature [23]. No recent prevalence estimates exist for males. OpenEpi software (v3.01) was employed to calculate the sample size.

### Instruments

The present study utilized a survey comprising two sections: one focused on socio-demographic information and the other on mental health issues (i.e., assessing depression, anxiety, and stress levels). The socio-demographic section comprised questions relating to age, marital status, residency, education level, economic status (high-income: more than $100 per month; middle-income: between $50 and $100 per month; low-income: less than $50 per month), cigarette smoking status, and whether participants had experienced a bad event in the past month (defined as any action or occurrence that occurred within the past month that led the participant to feel down or depressed). The interpretation of what constituted a *"bad event"* was left to the participants.

To assess participants' levels of depression, anxiety, and stress, the Persian version of the 21-item Depression, Anxiety and Stress Scale (DASS-21) was employed [26]. The scale is divided into three sub-domains: depression, anxiety, and stress. Responses for all items, such as *"I couldn't seem to experience any positive feeling at all"* are scored on a scale from 0 (*"Did not apply to me at all"*) to 3 (*"Applied to me very much or most of the time"*). Scores for each sub-domain range from 0 to 21. To obtain the final score for each sub-domain, the score was multiplied by two, resulting in the same scoring as the longer DASS-42. For the depression sub-domain, the standard cut-off scores were applied: 0 to 9 indicated normal levels, 10 to 13 indicated mild depression, 14 to 20 indicated moderate depression, and scores above 20 indicated severe to extremely severe depression symptoms. In the present study, Cronbach’s alpha for the depression subscale was 0.849. Similarly, for the anxiety sub-domain, scores between 0 and 7 are considered normal, 8 to 9 indicate mild anxiety, 10 to 14 indicate moderate anxiety, and scores higher than 14 indicate severe to extremely severe anxiety symptoms. Cronbach’s alpha for anxiety subscale in the present study was 0.836. For the stress sub-domain, scores between 0 and 14 are considered normal, 15 to 18 indicate mild stress, 19 to 25 indicate moderate stress, and scores higher than 25 indicate severe to extremely severe stress symptoms. Cronbach’s alpha for the stress subscale in the present study was 0.874.

### Analysis

The data were entered using Microsoft Excel 2016, while the analysis was conducted using IBM SPSS version 26.0 for Windows. Descriptive statistics comprised means, standard deviations, frequencies, and percentages. Associations between variables were assessed utilizing chi-square tests. To explore the independent socio-demographic factors associated with depression, anxiety, and stress, a multiple regression analysis was employed. Variables with a two-tailed *p*-value below 0.05 were considered statistically significant.

## Results

Of the 2698 participants, more than half of participants were female (54.3%), almost two-thirds of the participants were married (63.7%), and two-thirds of the participants were living in urban areas (66.6%). One-eighth of the participants had university level education (14.0%), and more than four-fifths of the participants had low-income economic status (81.0%). One-tenth had ever smoked, while nine-tenths of the participants had never smoked (90.2%). Table 1 provides a detailed breakdown of the participants’ characteristics (Table 1).

**Table 1:** Characteristics distribution of the study sample (N = 2698)

Characteristic	Categories	Male		Female		Total	
		n	%	n	%	n	%
Age group	15–34-years	914	47.5	1010	52.5	1924	71.3
	35–100-years	320	41.3	454	58.7	774	28.7
Marital status	Single	465	51.2	444	48.8	909	33.7
	Married	759	44.2	959	55.8	1718	63.7
	Widow/divorced	10	14.1	61	85.9	71	2.6
Residency	Urban	769	42.8	1029	57.2	1798	66.6
	Rural	465	51.7	435	48.3	900	33.4
Education	Illiterate	329	35.1	607	64.9	936	34.7
	Primary school	162	49.7	164	50.3	326	12.1
	Secondary school	242	51.4	229	48.6	471	17.4
	High school	324	55.1	264	44.9	588	21.8
	University	177	46.9	200	53.1	377	14.0
Economic status	High-income	54	59.3	37	40.7	91	3.4
	Middle-income	196	46.7	224	53.3	420	15.6
	Low-income	984	45.0	1203	55.0	2187	81.0
Cigarette smoking	Never smoked	1021	41.9	1413	58.1	2434	90.2
	Ex-smoker	120	76.4	37	23.6	157	5.8
	Current smoker	93	86.9	14	13.1	107	4.0
Bad event occurring in the past month	Yes	650	42.5	880	57.5	1530	56.7
	No	584	50.0	584	50.0	1168	43.3
Total		1234	100.0	1464	100.0	2698	100.0

The emboldened numbers simply emphasize the significant results

**Fig. 2:**
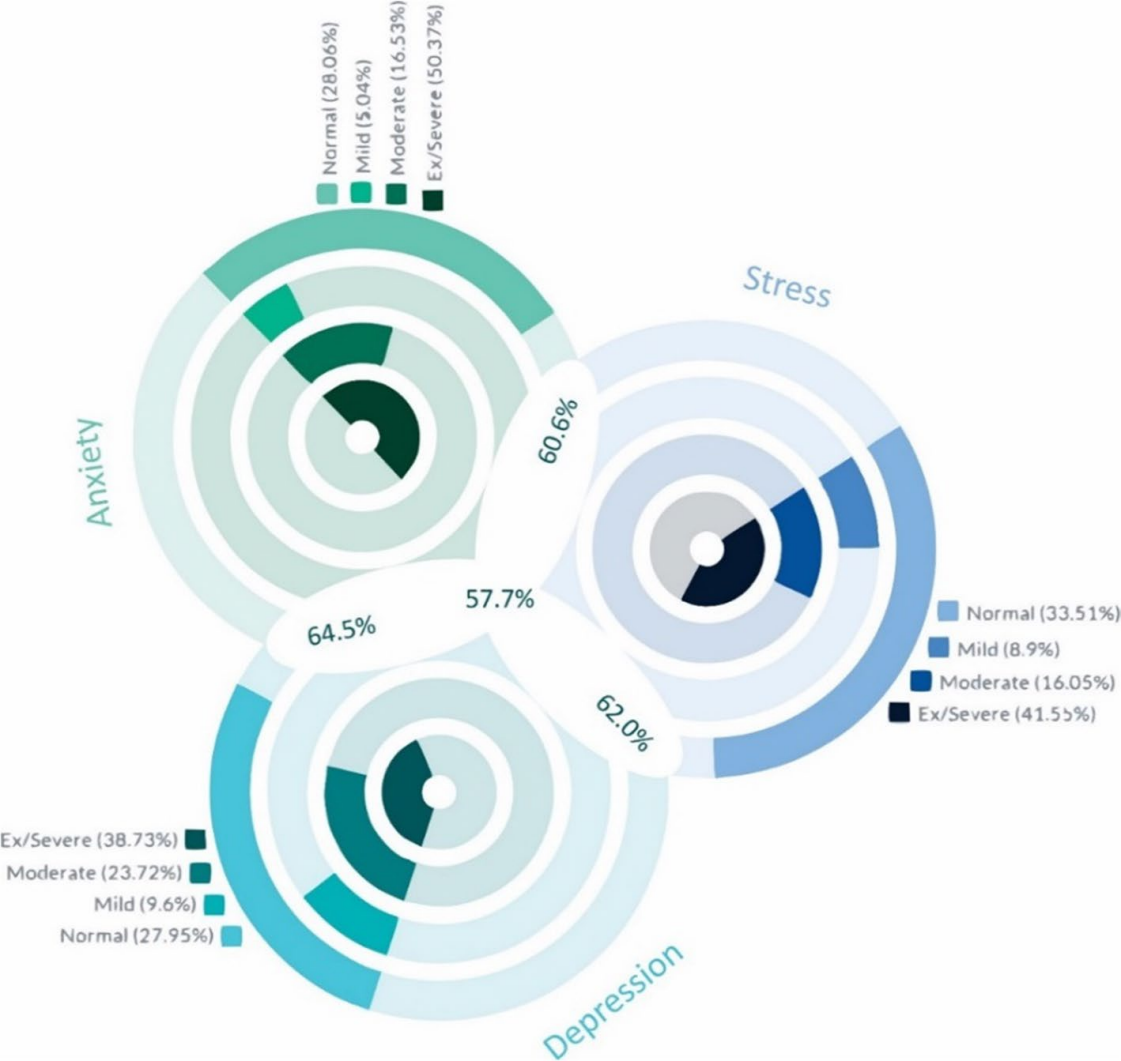
Categories of depression, anxiety, and stress (Afghanistan-2023)

Among the participants, 510 did not exhibit any signs of depression, anxiety, or stress (18.9%). Conversely, 1557 displayed symptoms of all three conditions (57.7%), with 50.3% reporting severe to extremely severe anxiety, 41.5% reporting severe to extremely severe stress, and 38.73% reporting severe to extremely severe depression. Figure 2 illustrates the intersecting prevalence of depression, anxiety, and stress among the study participants (Fig. 2).

Almost half of the participants aged 35–100-years had severe to extremely severe depression (49.7%), less than half of the female participants had severe to extremely severe depression (46.4%), and almost two-thirds of the widowed/divorced participants had severe to extremely severe depression (62.0%). More than one-third of the participants living in urban areas had severe to extremely severe depression (36.1%), and one-quarter of participants who had low economic status had no depression (24.4%). There was a significant relationship between presence of severe to extremely severe depression and (i) age group (more prevalent among older participants), (ii) gender (more prevalent among females), (iii) marital status (more prevalent among married and widow/divorced participants) (iv) residency (more prevalent among residents living in rural areas), (v) education (more prevalent among illiterate participants), (vi) economic status (more prevalent among participants with low-income economic status), and (vii) bad events (more prevalent among participants who experienced a bad event during the past month (Table 2).

**Table 2:** Association of depression with participants socio-demographic characteristics (n = 2698)

Characteristic	Depression				*p*-value
	Normal	Mild	Moderate	Severe and extremely severe	
	N (%)	N (%)	N (%)	N (%)	
Age group					
15–34-years	608 (31.6)	196 (10.2)	460 (23.9)	660 (34.3)	**< 0.001**
35–100-years	146 (18.9)	63 (8.1)	180 (23.3)	385 (49.7)	
Gender					
Male	426 (34.5)	146 (11.8)	297 (24.1)	365 (29.6)	**< 0.001**
Female	328 (22.4)	113 (7.7)	343 (23.4)	680 (46.4)	
Marital status					
Single	321 (35.3)	105 (11.6)	226 (24.9)	257 (28.3)	**< 0.001**
Married	422 (24.6)	150 (8.7)	402 (23.4)	744 (43.3)	
Widow/divorced	11 (15.5)	4 (5.6)	12 (16.9)	44 (62.0)	
Residency					
Urban	554 (30.8)	172 (9.6)	423 (23.5)	649 (36.1)	**< 0.001**
Rural	200 (22.2)	87 (9.7)	217 (24.1)	396 (44.0)	
Education					
Illiterate	157 (16.8)	73 (7.8)	200 (21.4)	506 (54.1)	**< 0.001**
Primary school	77 (23.6)	26 (8.0)	90 (27.6)	133 (40.8)	
Secondary school	133 (28.2)	57 (12.1)	116 (24.6)	165 (35.0)	
High school	231 (39.3)	62 (10.5)	143 (24.3)	152 (25.9)	
University	156 (41.4)	41 (10.9)	91 (24.1)	89 (23.6)	
Economic status					
High-income	51 (56.0)	5 (5.5)	18 (19.8)	17 (18.7)	**< 0.001**
Middle-income	170 (40.5)	48 (11.4)	110 (26.2)	92 (21.9)	
Low-income	533 (24.4)	206(9.4)	512 (23.4)	936 (42.8)	
Cigarette smoking					
Never smoked	695 (28.6)	226 (9.3)	570 (23.4)	943 (38.7)	0.265
Ex-smoker	38 (24.2)	19 (12.1)	42 (26.8)	58 (36.9)	
Current smoker	21 (19.6)	14 (13.1)	28 (26.2)	44 (41.1)	
Bad event occurring in the past month					
Yes	315 (20.6)	146 (9.5)	367 (24.0)	702 (45.9)	**< 0.001**
No	439 (37.6)	113 (9.7)	273 (23.4)	343 (29.4)	
Total	754 (27.9)	259 (9.6)	640 (23.7)	1045 (38.7)	

The emboldened numbers simply emphasize the significant results

Almost two-thirds of the participants aged 35–100-years had severe to extremely severe anxiety (66.9%), more than half of the female participants had severe to extremely severe anxiety (56.8%), and almost three-quarters of the widowed/divorced participants had severe to extremely anxiety (71.8%). More than half of the participants living in rural areas had severe to extremely severe anxiety (57.1%), and one-quarter who had low economic status had no anxiety (24.3%). There was a significant relationship between presence of severe/extremely severe anxiety and (i) age (more prevalent among older participants), (ii) gender (more prevalent among females), (iii) marital status (more prevalent among married and widow/divorced participants) (iv) residency (more prevalent among residents living in rural areas), (v) education (more prevalent among illiterate participants), (vi) economic status (more prevalent among participants with low-income economic status), and (vii) bad events (more prevalent among participants who experienced a bad event during the past month (Table 3).

**Table 3:** Association of anxiety with participants socio-demographic characteristics (n = 2698)

Characteristic	Anxiety				*p*-value
	Normal	Mild	Moderate	Ex/Severe	
	N (%)	N (%)	N (%)	N (%)	
Age group					
15–34-years	640 (33.3)	106 (5.5)	337 (17.5)	841 (43.7)	**< 0.001**
35–100-years	117 (15.1)	30 (3.9)	109 (14.1)	518 (66.9)	
Gender					
Male	429 (34.8)	63 (5.1)	215 (17.4)	527 (42.7)	**< 0.001**
Female	328 (22.4)	73 (5.0)	231 (15.8)	832 (56.8)	
Marital status					
Single	352 (38.7)	53 (5.8)	160 (17.6)	344 (37.8)	**< 0.001**
Married	395 (23.0)	82 (4.8)	277 (16.1)	964 (56.1)	
Widow/divorced	10 (14.1)	1 (1.4)	9 (12.7)	51 (71.8)	
Residency					
Urban	565 (31.4)	90 (5.0)	298 (16.6)	845 (47.0)	**< 0.001**
Rural	192 (21.3)	46 (5.1)	148 (16.4)	514 (57.1)	
Education					
Illiterate	122 (13.0)	30 (3.2)	145 (15.5)	639 (68.3)	**< 0.001**
Primary school	77 (23.6)	13 (4.0)	64 (19.6)	172 (52.8)	
Secondary school	137 (29.1)	42 (8.9)	76 (16.1)	216 (45.9)	
High school	252 (42.9)	34 (5.8)	93 (15.8)	209 (35.5)	
University	169 (44.8)	17 (4.5)	68 (18.0)	123 (32.6)	
Economic status					
High-income	48 (52.7)	3 (3.3)	13 (14.3)	27 (29.7)	**< 0.001**
Middle-income	178 (42.4)	30 (7.1)	73 (17.4)	139 (33.1)	
Low-income	531 (24.3)	103 (4.7)	360 (16.5)	1193 (54.5)	
Cigarette smoking					
Never smoked	691 (28.4)	120 (4.9)	405 (16.6)	1218 (50.0)	0.819
Ex-smoker	42 (26.8)	9 (5.7)	24 (15.3)	82 (52.2)	
Current smoker	24 (22.4)	7 (6.5)	17 (15.9)	59 (55.1)	
Bad event occurring in the past month					
Yes	315 (20.6)	83 (5.4)	268 (17.5)	864 (56.5)	**< 0.001**
No	442 (37.8)	53 (4.5)	178 (15.2)	495 (42.4)	
Total	757 (28.1)	136 (5.0)	446 (16.5)	1359 (50.4)	

The emboldened numbers simply emphasize the significant results

More than half of the participants aged 35–100-years had severe to extremely severe stress (55.0%), more than half of the females had severe to extremely severe stress (52.6%), and almost one-third of the widowed/divorced participants had severe to extremely severe stress (62.0%). Less than half of the participants living in rural areas had severe to extremely stress (44.3%), and more than one-quarter of the participants who had low economic status had no stress (29.6%). There was a significant relationship between presence of severe/extremely severe stress and (i) age (more prevalent among older participants), (ii) gender (more prevalent among females), (iii) marital status (more prevalent among married and widow/divorced participants) (iv) residency (more prevalent among residents living in rural areas), (v) education (more prevalent among illiterate participants), (vi) economic status (more prevalent among participants with low-income economic status), and (vii) bad events (more prevalent among participants who experienced a bad event during the past month (Table 4).

**Table 4:** Association of stress with participants socio-demographic characteristics (n = 2698)

Characteristic	Stress				*p*-value
	Normal	Mild	Moderate	Ex/Severe	
	N (%)	N (%)	N (%)	N (%)	
Age group					
15–34-years	737 (38.3)	197 (10.2)	295 (15.3)	695 (36.1)	**< 0.001**
35–100-years	167 (21.6)	43 (5.6)	138 (17.8)	426 (55.0)	
Gender					
Male	545 (44.2)	125 (10.1)	213 (17.3)	351 (28.4)	**< 0.001**
Female	359 (24.5)	115 (7.9)	220 (15.0)	770 (52.6)	
Marital status					
Single	399 (43.9)	108 (11.9)	152 (16.7)	250 (27.5)	**< 0.001**
Married	488 (28.4)	129 (7.5)	274 (15.9)	827 (48.1)	
Widow/divorced	17 (23.9)	3 (4.2)	7 (9.9)	44 (62.0)	
Residency					
Urban	641 (35.7)	180 (10.0)	255 (14.2)	722 (40.2)	**< 0.001**
Rural	263 (29.2)	60 (6.7)	178 (19.8)	399 (44.3)	
Education					
Illiterate	168 (17.9)	52 (5.6)	144 (15.4)	572 (61.1)	**< 0.001**
Primary school	103 (31.6)	27 (8.3)	63 (19.3)	133 (40.8)	
Secondary school	167 (35.5)	37 (8.3)	82 (17.4)	185 (39.3)	
High school	281 (47.8)	37 (7.9)	86 (14.6)	150 (25.5)	
University	185 (49.1)	71 (12.1)	58 (15.4)	81 (21.5)	
Economic status					
High-income	58 (63.7)	8 (8.8)	8 (8.8)	17 (18.7)	**< 0.001**
Middle-income	199 (47.4)	50 (11.9)	73 (17.4)	98 (23.3)	
Low-income	647 (29.6)	182 (8.3)	352 (16.1)	1006 (46.0)	
Cigarette smoking					
Never smoked	822 (33.8)	210 (8.6)	368 (15.1)	1034 (42.5)	**0.001**
Ex-smoker	51 (32.5)	19 (12.1)	41 (26.1)	46 (29.3)	
Current smoker	31 (29.0)	11 (10.3)	24 (22.4)	41 (38.3)	
Bad event occurring in the past month					
Yes	396 (25.9)	131 (8.6)	253 (16.5)	750 (49.0)	**< 0.001**
No	508 (43.5)	109 (9.3)	180 (15.4)	371 (31.8)	
Total	904 (33.5)	240 (8.9)	433 (16.0)	1121 (41.5)	

The emboldened numbers simply emphasize the significant results

Multiple logistic regression analysis was performed to see which variables predicted depression, anxiety, and stress. The variables that were significantly associated with depression were: gender (being female) (AOR = 1.762, *p* < 0.001), economic status (having a low-income) (AOR = 2.627, *p* < 0.001), residency (living in a rural area) (AOR = 1.233, *p* = 0.043), education (being illiterate) (AOR = 0.345, *p* < 0.001), cigarette smoking (current-smoker) (AOR = 2.785, *p* < 0.001) and a bad event occurring in the past month (AOR = 0.436, *p* < 0.001). The variables that were significantly associated with anxiety were: age (being older) (AOR = 1.526, *p* < 0.001), gender (being female) (AOR = 1.716, *p* < 0.001), economic status (having a low-income) (AOR = 2.080, *p* = 0.002), residency (living in a rural area) (AOR = 1.262, *p* = 0.029), education (being illiterate) (AOR = 0.237, *p* < 0.001), cigarette smoking (current-smoker) (AOR = 2.496, *p* < 0.001) and a bad event occurring in the past month (AOR = 0.412, *p* < 0.001). The variables that were significantly associated with stress were: gender (being female) (AOR = 2.353, *p* < 0.001), economic status (having a low-income) (AOR = 2.747, *p* < 0.001), education (being illiterate) (AOR = 0.265, *p* < 0.001), cigarette smoking (current smoker) (AOR = 2.521, *p* < 0.001), and a bad event occurring in the past month (AOR = 0.449, *p* < 0.001) (Table 5).

**Table 5:** Multiple logistic regression analysis of depression, anxiety and stress on participants’ sociodemographic characteristics in Afghanistan (n = 2698)

Variable	Depression		Anxiety		Stress	
	AOR [95% CI]	*p*-value	AOR [95% CI]	*p*-value	AOR [95% CI]	*p-*value
Age group						
15–34-years	Reference		Reference		Reference	
35–100-years	1.211 [0.950, 1.543]	0.122	1.526 [1.182, 1.972]	**0.001**	1.250 [0.990, 1.578]	0.061
Gender						
Male	Reference		Reference		Reference	
Female	1.762 [1.463, 2.123]	**< 0.001**	1.716 [1.419, 2.074]	**< 0.001**	2.353 [1.963, 2.820]	**< 0.001**
Economic status						
High-income	Reference		Reference		Reference	
Middle-income	1.586 [1.257, 2.002]	**< 0.001**	1.618 [1.279, 2.047]	**0.002**	1.613 [1.281, 2.031]	**< 0.001**
Low-income	2.627 [1.669, 4.136]	**< 0.001**	2.080 [1.315, 3.292]	**< 0.001**	2.747 [1.717, 4.395]	**< 0.001**
Residency						
Urban	Reference		Reference		Reference	
Rural	1.233 [1.007, 1.511]	**0.043**	1.262 [1.025, 1.554]	**0.029**	1.042 [0.858, 1.265]	0.678
Education						
Illiterate	Reference		Reference		Reference	
Primary school	0.913 [0.691, 1.205]	0.519	0.904 [0.686, 1.190]	0.471	0.923 [0.701, 1.215]	0.568
Secondary school	0.593 [0.438, 0.802]	**0.001**	0.524 [0.388, 0.708]	**< 0.001**	0.577 [0.430, 0.775]	**< 0.001**
High school	0.466 [0.330, 0.659]	**< 0.001**	0.403 [0.285, 0.570]	**< 0.001**	0.488 [0.351, 0.679]	**< 0.001**
University	0.345 [0.254, 0.469]	**< 0.001**	0.237 [0.173, 0.324]	**< 0.001**	0.265 [0.196, 0.358]	**< 0.001**
Cigarette smoking						
Never smoked	Reference		Reference		Reference	
Ex-smoker	1.724 [0.919, 3.237]	0.090	1.790 [0.970, 3.304]	0.063	1.632 [0.923, 2.885]	0.092
Current smoker	2.785 [1.663, 4.666]	**< 0.001**	2.496 [1.514, 4.115]	**< 0.001**	2.521 [1.589, 3.999]	**< 0.001**
Bad event occurring in past month						
Yes	Reference		Reference		Reference	
No	0.436 [0.364, 0.523]	**< 0.001**	0.412 [0.343, 0.496]	**< 0.001**	0.449 [0.376, 0.536]	**< 0.001**

The emboldened numbers simply emphasize the significant results

## Discussion

The present survey was conducted to investigate the mental health challenges experienced by the Afghan populace and ascertain the socio-demographic variables correlated with depression, anxiety, and stress. The mental health of Afghans living under Taliban rule is a matter of the utmost importance, and it is essential to understand some of the complex factors contributing to this issue. The Taliban's return to power in Afghanistan has brought about significant political, social, and economic changes that appear to have had a profound impact on the well-being of the Afghan population [27, 28]. Several studies have reported severe anxiety, depression and stress among the Afghan people but these have been localized studies and/or included a specific cohort of Afghan society [22, 23, 29]. The survey was carried out to examine the mental health issues faced by Afghan people and determine the socio-demographic factors associated with stress, anxiety, and depression. In the present study, less than one-fifth of the participants (18.9%) did not exhibit any signs of depression, anxiety, or stress.

In the present study, approximately one-fifth of the participants (27.9%) indicated a mental state free of any depression symptoms, with females exhibiting a higher prevalence of depression symptoms (77.6%) than males (65.5%). Consistent with previous research, gender emerged as a significant factor influencing mental health status, with females manifesting elevated levels of mental health disorders compared to males [23, 30]. The findings indicated that 64.5% of those reporting depression symptoms also reported anxiety symptoms. Moreover, 62.0% of individuals reporting depression symptoms also reported experiencing stress. Factors significantly associated with depression included age (being older), gender (being female), marital status (being widow/divorced), residency (residing in rural areas), education level (having lower educational attainment), economic status (having a low monthly income), cigarette smoking (being a current smoker), and experiencing a bad event in the past month.

The prevalence of depression symptoms in the present study was much higher than the reported range by the World Health Organization (WHO), which indicates a frequency of 1 in 10 individuals in areas affected by conflict. However, the present study found a markedly elevated prevalence of nearly 7 in 10 individuals, greatly surpassing the figures by the WHO [15]. Those aged over 34 years, exhibited a much higher prevalence of depression symptoms (81.1%) compared to the younger age group aged under 35 years (68.4%). In comparison, data from a 2019 interview survey conducted by the Centers for Disease Control and Prevention (CDC) in the US indicated that 21.0% of adults aged 18–29 years experienced depression symptoms, compared to 16.8% among adults aged 30–44 years [31].

The disparity in depression symptoms between age groups may reflect differences in life stressors, financial burdens, and social isolation, with older individuals potentially facing more complex challenges. The findings of this study align with a previous study which reported 79.0% depression among women in Afghanistan (79.0%) [22]. This suggests that depression symptoms remained the same even after the war ended in Afghanistan. This finding is consistent with another study which found that psychological distress symptoms were prevalent among 75% of the national Afghan population [32], but much higher than a study among Afghan pregnant women which reported the prevalence of depression to be 60.9% [33], Compared to countries elsewhere in the world, a systematic review by Mahmud et al. [34] examining depression during the COVID-19 pandemic indicated that 30.5% of the global population exhibited symptoms of depression. These findings suggest that in Afghanistan, the prevalence of depression exceeds that of the global average. Notably, under the Taliban government, there appears to have been a slight increase in this prevalence.

Moreover, in the present study, the presence of depression symptoms was significantly associated with educational attainment, with illiterate participants reporting a higher prevalence (83.2%) compared to those with a university education (58.6%). This is in line with findings of previous study in Afghanistan that reported a significant association between educational level and presence of depression symptoms [23]. This finding also aligns with a European study that reported higher levels of education being associated with lower odds of depression [35]. A meta-analysis further supports the crucial role of educational levels in shaping mental health outcomes, including depression, showing that those with lower education have poorer mental health outcomes [36].

Almost three-quarters of participants reported anxiety symptoms (71.9%) with those aged over 34 years reporting a higher prevalence (84.9%), in contrast to those aged under 35 years (66.7%). This is in line with findings of a previous study among women in Afghanistan (n = 438) which reported that the prevalence of anxiety among older participants (89.1%) was higher than younger ones (75.6%) [23], a finding that is generally supported in the literature [22, 23]. This differs from findings in a study conducted in Iran (n = 7886), where the prevalence of anxiety was reported to be higher among younger participants (20.1%) than older participants (13.8%) [37]. Additionally, a study conducted in Malaysia (n = 506) reported higher levels of anxiety in the younger age group (9.61%) compared to old age groups (12.8%) [38]. A meta-analysis by Mahmud et al. reported that 29.6% of the world population during the COVID-19 pandemic had anxiety [34]. This suggests that compared to the rest of the world, anxiety appears to be more prevalent in Afghanistan. The different rate observed in the present study’s findings compared to other studies may be due to cultural, geographic, and methodological differences, impacting the manifestation and disclosure of anxiety symptoms across distinct demographic cohorts and locales.

Marital status emerged as another significant factor associated with anxiety symptoms, with widowed/divorced participants reporting the highest percentage of anxiety (85.9%) compared to the other two groups (61.3% single and 77.0% married). This aligns with prior systematic reviews indicating that being divorced or widowed are significant predictors of anxiety among women [35, 39]. This is in line with previous study in Afghanistan (n = 438) which reported higher prevalence of anxiety among divorced participants compared to single and married participants [23].

With regards to educational attainment, participants with at least a university-level education reported the lowest levels of anxiety (55.2%), while a significantly higher proportion of illiterate participants reported anxiety symptoms (87.0%). This observation aligns with a previous study in India (n = 180) indicating an increased likelihood of anxiety among women with lower educational attainment [40]. Moreover, in the present study, specific demographic subgroups reported elevated anxiety levels compared to their counterparts. More specifically, those with a low family monthly income recorded a higher prevalence of anxiety (75.7%) in comparison to those with a high family monthly income (47.3%). This finding is in line with the findings of a previous study in Afghanistan (n = 664) that reported higher prevalence of anxiety among low monthly family income participants compared to high-income monthly family income [22]. Additionally, individuals who experienced a bad event in the past month reported higher anxiety levels (79.4%) compared to those who did not (62.2%). These findings align with the findings of previous aforementioned study in Afghanistan that reported higher prevalence of anxiety among participants who experienced bad event in the past month than those who had not [23].

Over half of participants aged over 34 years (55%) reported symptoms of stress. The study found significant associations between various demographic factors and stress, including gender, age group, marital status, residency, traumatic experiences, and economic status. More specifically, the findings indicated that older individuals, females, and those with lower income levels reported more stress than their counterparts. These findings are novel as no previous study in Afghanistan has assessed the prevalence of stress among the general population. However, studies have been conducted on the mental health of healthcare workers [41], adolescents [42], and women in Afghanistan [22]. These results diverge from those of a systematic meta-analytic review conducted by Salari et al. among general population [43], which identified a negative association between stress and the age group of participants (n = 9074).

In the present study, prevalence of stress was 66.49%. This is much higher than the findings of two systematic meta-analytic reviews by Mahmud et al. [34] and Salari et al. [43] who reported that during the COVID-19 pandemic, the prevalence of stress was 29.4% and 29.6% globally, respectively [34]. In the present study, a higher prevalence of stress was observed among married participants (71.6%) compared to their single counterparts (56.1%). This contrasts with findings in a study by Cao et al. in China [44], where prevalence of stress among single marital status was higher than married participants (n = 9030). Moreover, the impact of traumatic experiences on individuals' lives cannot be understated [45], and the findings of the present study reinforce existing evidence that individuals with a history of traumatic events are more susceptible to the development of stress [29, 46, 47]. These findings underscore the intricate interplay between demographic variables, life experiences, and stress manifestation, contributing valuable insights to the understanding of stress dynamics within diverse populations.

Multiple regression analysis indicated that gender (being female), economic status (being poor), residency (living in rural areas), education level (being illiterate), being a cigarette smoker, and having experienced a bad event during the past month were significantly associated with depression, anxiety and stress. These findings underscore the interplay of these demographic and behavioral factors in influencing the manifestation of mental health symptoms. Importantly, extant literature has consistently corroborated the significant impact of depression on these specific demographic factors, as evidenced by prior studies [48–51], further affirming the robustness of the present study’s findings. The study’s findings also indicate the multifaceted nature of the possible determinants of anxiety symptoms, providing insight into the interconnectedness of various demographic and behavioral elements. Notably, existing research has consistently highlighted the substantial impact of anxiety on the aforementioned demographic factors, as substantiated by many prior investigations [52–58].

A significant proportion of cigarette smokers in the present study (80.4%) exhibited indications of depression symptoms. Additionally, 77.6% of current smokers displayed symptoms indicative of anxiety, while 71.0% reported experiencing symptoms associated with stress. Current smokers exhibited a prevalence of depression symptoms 2.8 times greater than individuals who had never smoked. Similarly, they displayed a prevalence of anxiety symptoms 2.5 times higher and a prevalence of stress symptoms 2.5 times higher compared to individuals who had never smoked. These findings are in line with results of a systematic review conducted by Fluharty et al. which reported that smoking was associated with depression and anxiety [560]. Other systematic reviews have reported that smoking is associated with depression [59] and anxiety [60], and that the relationships are bi-directional, and that one can lead to another [59, 60].

It is noteworthy that the present study’s findings resonate with broader trends observed in the national Afghan landscape. A study conducted in major provinces of Afghanistan in 2021 (n = 664) reported that almost four-fifths of Afghan women exhibited depression symptoms (79.1%) [22]. This alignment of findings suggests an increase in high prevalence of mental health disorders among the Afghan population.

## Limitations

The present study had a number of limitations. Firstly, the study lacked an assessment of the onset dates of depression, anxiety, and/or stress, precluding the determination of whether participants' mental health conditions changed post-Taliban takeover or had pre-existing origins. Notably, the reliance on self-reported data introduces potential methodological biases, despite providing estimates of depression, anxiety, and stress among the Afghan population and their associations with socio-demographic factors. The cross-sectional design of the study further restricts the capacity to determine causality between the examined variables. Furthermore, the non-representative nature of the sample is a limitation because it encompasses participants from only three regions in Afghanistan, with an overrepresentation of urban residents compared to the national distribution. Consequently, the sample exhibits a disproportionately lower proportion of illiterate participants than the national demographic composition, undermining the generalizability of the study's findings to all Afghans. The scarcity of previous investigations into the mental health of the general Afghan population since the resurgence of the Taliban hindered the ability to contextualize and compare the results of the present study with other studies, limiting the discernment of meaningful trends. Future studies should use longitudinal designs, objective assessments, and larger, more representative samples, including diverse regions and comparing with similar studies will provide better context. Employing causal analysis would clarify socio-demographic impacts on mental health, improving research accuracy and depth.

## Conclusion and recommendations

The mental health crisis in Afghanistan is a complex and urgent issue that requires a comprehensive and compassionate response. The findings of the present study showed very high levels of anxiety, depression, and stress among the participants. The study offered important insights into the mental health status of Afghans living under the Taliban government. The high prevalence rates of depression, anxiety, and stress underscore the substantial psychological burden experienced by the population. The identified socio-demographic factors, such as being female, being of low economic status, living in rural regions, having a low education level, being a cigarette smoker, and recent traumatic experiences, highlight the complexity of mental health disparities in this context. These findings emphasize the pressing need for targeted and culturally sensitive interventions to address the multifaceted challenges contributing to the high prevalence of mental health disorders. Tailored mental health programs, informed by the findings here, could play a pivotal role in mitigating the impact of psychological distress and promoting overall well-being among Afghans living under the current sociopolitical circumstances.

Given the high rates of mental health issues among Afghans under Taliban rule, urgent targeted interventions are needed. Policymakers should focus on community-based programs, training local professionals, and integrating traditional healing with evidence-based therapies. Collaboration with international organizations is key to building a sustainable mental health infrastructure.

The findings of the present study suggest a potential comorbidity between mental health disorders and various socio-demographic vulnerabilities among Afghans, necessitating urgent attention to address the prevailing mental health challenges under the current Taliban governance. Subsequent research endeavors should explore the origins of therapeutic resources and assess their accessibility within the broader Afghan population. Moreover, as the international community engages with Afghanistan, it must prioritize the well-being of the Afghan individuals and provide the necessary resources to address their mental health needs. This includes providing funding for mental health services, training local mental health professionals, and raising awareness about mental health issues to reduce stigma.

## Data Availability

All data relevant to the study are included in the article or uploaded as supplementary information. The Dari version of the DASS-21 questionnaire (preprint) used in this study can be accessed at the following link: https://www.researchsquare.com/article/rs-4337555/latest.
